# 한국 중년 폐경여성의 대사증후군과 자율신경활성도 연관성: 횡단적 조사 연구

**DOI:** 10.7586/jkbns.25.051

**Published:** 2025-11-11

**Authors:** Myung-hwa Kim, So-Eun Choi

**Affiliations:** 1 Wonkwang University, Jangheung Integrative Medical Hospital, Jangheung-gun, Jeollanam-do, Korea; 1 원광대학교 장흥통합의료병원; 2 Department of Nursing, Mokpo National University, Muan-gun, Jeollanam-do, Korea; 2 국립목포대학교 간호학과

**Keywords:** Postmenopause, Middle aged, Women, Metabolic syndrome, Autonomic nervous system, 폐경, 중년, 여성, 대사증후군, 자율신경계

## Abstract

**Purpose:**

This study aimed to investigate the association between metabolic syndrome and autonomic nervous system activity in middle-aged postmenopausal women. In addition, it explored the clinical applicability of heart rate variability (HRV) as a physiological marker for the early diagnosis and management of metabolic disorders.

**Methods:**

This study was conducted as a secondary data analysis using health examination data. The dataset included 276 postmenopausal women aged 50 to 65 years. Autonomic nervous system activity was evaluated using HRV indices, including standard deviation of the normal-to-normal interval (SDNN), total power (TP), low frequency (LF), and high frequency (HF). Statistical analyses were performed using the t-test, chi-square test, and Pearson correlation analysis with SPSS version 26.0.

**Results:**

The prevalence of metabolic syndrome among the participants was 21.4%. The LF index was significantly lower in the metabolic syndrome group compared with the normal group (t = 2.74, *p* = .007). Additionally, fasting blood glucose showed significant negative correlations with SDNN (r = -.16, *p* = .010), TP (r = −.21, *p* < .001), LF (r = −.17, *p* = .004), and HF (r = −.18, *p* = .003).

**Conclusion:**

A reduction in HRV, particularly in LF, may serve as an indicator of metabolic syndrome in postmenopausal women. Because autonomic dysfunction can potentially be improved through nursing interventions, the use of HRV-based monitoring and personalized nursing strategies should be considered in clinical practice to help prevent the onset of metabolic syndrome in postmenopausal women.

## 서론

### 1. 연구의 필요성

중년기는 생리학적 노화가 시작되는 시기로 사회적, 심리적 변화와 함께 신체기능에 많은 변화가 나타나 건강관리가 매우 중요한 시기이다. 특히 여성의 경우 대부분 49세에서 52세 사이에 폐경에 이르는데, 중년여성의 건강을 개선하기 위해서는 폐경 증상을 이해하고 관리하는 것이 중요하다[1]. 여성은 폐경 이후 여성호르몬인 에스트로겐이 급격히 감소하면서 여러 신체적 변화를 경험하게 되며, 이로 인해 혈중 지질대사의 변화, 복부 지방의 축적에 따른 내장 비만, 인슐린 저항성 증가 등이 나타나 심혈관계 질환 및 대사성 질환의 발병 위험이 높아지는 것으로 알려져 있다[2,3]. 실제로 폐경 이후 여성의 대사증후군 이환율은 폐경 전보다 약 3배 이상 급격히 증가하며[4] 에스트로겐 저하에 따른 혈관 기능 저하와 지방 대사 이상은 대사성질환 발병 위험을 60% 정도 증가시키는 것으로 보고되어[5] 중년기 폐경 여성에게 있어 대사증후군 예방 및 관리가 매우 중요함을 시사한다.

대사증후군은 복부비만, 고혈압, 고혈당, 이상지질혈증 등이 동시에 생기는 일종의 질환군으로 복부둘레, 혈압, 공복혈당, 고밀도지단백 콜레스테롤, 중성지방 중 3가지 이상에서 비정상인 경우 대사증후군으로 정의한다[6]. 대한비만학회에서 발표한 자료에 따르면 우리나라 성인의 대사증후군 유병률은 2013년 23.3%에서 최근 10년 사이 2022년 기준으로 28.6%로 증가하였다[7]. 대사증후군 위험요인을 1∼2개 가지고 있는 주의군도 2018년 기준 45.5%나 되는 것으로 보고되고 있다[8]. 최근 대사증후군과 관련하여 그 원인으로 자율신경계 불균형이 주요 이슈로 제기되고 있어[9], 자율신경이상검사가 비침습적이면서도 안전한 검사[10]로 주목받고 있다.

자율신경계는 교감, 부교감, 장신경계로 구성되어 신체 전반에 분포하며 자율신경 반사를 통해 혈압, 심박수, 위장관, 방광, 성기능, 체온, 호흡 등을 조절하여 신체 항상성을 유지하고 조절하는 역할을 한다[11,12]. 자율신경계는 스트레스에 반응하여 교감신경과 부교감신경의 균형을 조절하며, 이 균형이 깨질 경우 대사 이상을 초래할 수 있다[13]. 특히 폐경기 여성은 호르몬 변화로 인해 자율신경계의 불균형이 심화될 수 있으며 이는 대사증후군 발병과 밀접한 관련이 있다[14]. 선행연구에서도 자율신경불균형이 대사증후군의 위험도를 증가시킬 수 있다고 하였다[15]. 이와 함께 최근에는 자율신경계의 기능을 반영하는 생리적 지표인 심박변이도(heart rate variability, HRV)가 대사질환 및 심혈관질환의 위험 예측에 중요한 역할을 할 수 있다는 연구들이 제시되고 있다[15-17]. HRV 검사는 시간에 따라 변화하는 심장박동 간 변동을 측정하여 분석하는 방법으로 시간 영역 분석(time domain analysis)과 주파수 영역 분석(frequency domain analysis)이 있다. 시간 영역 지표에는 정상 심박 간격(normal to normal interval)의 표준편차를 나타내는 SDNN (standard deviation of the normal to normal interval)이 있다. 주파수 영역은 시계열 데이터를 주파수 분석(power spectral analysis)을 통해 얻어지며, 주요 지표로는 VLF (very low frequency), LF (low frequency), HF (high frequency) 등이 있다[18,19]. 이중 LF는 교감신경을, HF는 부교감신경을 반영한다[15]. HRV 검사는 1996년 유럽 심장학회와 미국 심장학회 태스크 포스에 의해 자율신경 조절기능을 평가하는 유용한 비침습적인 지표로 인정되었으며, 심혈관계 질환의 예측인자이자 스트레스를 포함한 자율신경 이상과 관련된 다양한 질환 진단인자로 활용되고 있다[19].

국내에서 시행된 선행연구를 살펴보면 대학병원의 건강검진을 받은 성인 환자를 대상으로 한 연구에서 비만환자에서 인슐린 저항성을 매개로 자율신경활성도의 저하 및 자율신경계 불균형이 유발되며, 미주신경 기능 저하와 함께 대사증후군 위험이 유의하게 증가한다고 보고되었다[15]. 또다른 연구에서는 건강검진을 받은 성인 남녀를 구분하여 분석한 결과, 자율신경활성도가 저하된 여성에서 복부비만과 이상지질형증 위험이 더 높다고 하였다[20]. 이처럼 국내 선행연구는 대부분 일반 성인 남녀를 대상으로 진행되었으며, 연령 범위도 20대부터 80대까지 다양하였으나 폐경 여성만을 특정 대상으로 한 연구는 없었다. 한편 국외 연구에서는 치료받지 않은 갑상선기능항진증 환자와 건강한 대조군 간의 자율신경활성도 지표를 비교한 22편의 문헌을 통해, 갑상선 호르몬의 영향으로 자율신경활성도가 감소하는 경향이 확인되었으며[21], 불안장애 환자를 대상으로 한 99편의 메타분석에서는 건강인에 비해 부교감 신경활동이 유의하게 낮은 것으로 보고되었다[22]. 그러나 2023년 한국보건의료원 보고서[10]에 따르면 현재까지 국내에서 자율신경활성도와 특정 질환과의 관련성을 분석한 연구는 소아 두개인두종 질환과 성인 대사증후군을 대상으로 한 일부 연구[15]에 한정되며, 특히 폐경기 여성의 대사증후군과 자율신경활성도 간의 연관성을 구체적으로 분석한 연구는 보고된 바 없다.

이에 본 연구는 중년 폐경기 여성의 대사증후군과 자율신경활성도의 연관성을 규명하여, 폐경기 여성의 건강관리에 있어 자율신경 기반 생리적 지표의 활용 가능성을 제시하고, 폐경기 여성의 맞춤형 건강관리 및 간호중재 개발을 위한 기초자료를 제공하고자 한다.

### 2. 연구의 목적

중년기 폐경 여성을 대상으로 대사증후군과 자율신경활성도의 관련성을 파악하기 위함이며 구체적인 목적은 다음과 같다.

첫째, 중년기 폐경 여성의 대사증후군 유무에 따른 일반적 특성과 건강관련 특성을 파악한다.

둘째, 중년기 폐경 여성의 대사증후군 유무에 따른 대사증후군 위험요인을 파악한다.

셋째, 중년기 폐경 여성의 대사증후군 유무에 따른 자율신경활성도 차이를 파악한다.

넷째, 중년기 폐경 여성의 대사증후군 위험요인과 자율신경활성도의 상관관계를 확인한다.

## 연구 방법

### 1. 연구 설계

본 연구는 중년기 폐경 여성을 대상으로 한 대사증후군과 자율신경활성도의 관련성을 파악하기 위해 일개 병원에 등록된 자료를 분석한 이차자료 분석 연구이다.

### 2. 연구 대상

본 연구 대상자는 전남 장흥군에 위치한 원광대학교 장흥통합의료병원에 2023년 8월 1일부터 2024년 12월 31일까지 검진목적으로 내원하여 채혈검사, 복부둘레, 자율신경검사 등의 검진을 시행한 전체 중년기 여성 309명 중 폐경 여성 전수 276명을 대상으로 하였다. 본 연구의 표본 수가 적절한지 확인하기 위해 G*Power (version 3.1.9.2, Heinrich-Heine-Universität Düsseldorf, Düsseldorf, Germany)을 이용하여 사후 분석하였다. 본 연구의 상관관계 계수 .21, 유의수준 .05, 표본 수 276명일 때, 통계적 검정력은 .94로 표본 수가 충분하고 적절하다고 판단된다.

### 3. 연구 도구

#### 1) 일반적 특성

일반적 특성은 한국 성인을 대상으로 대사증후군과 관련된 요인을 보고한 선행연구[23]를 참고하여 자료에서 획득할 수 있는 변수 중 일반적 특성 및 건강 관련 특성을 선정하였다. 연령, 교육수준, 결혼상태, 흡연, 음주, 운동실천을 포함하였다. 교육정도는 졸업을 기준으로 초등학교 졸업 이하, 중학교 졸업, 고등학교 졸업, 대학교 졸업 이상으로 구분하고, 결혼상태는 배우자와 동거중인 사람과 미혼, 이혼, 사별 등으로 배우자와 동거하지 않는 경우로 구분하였다. 흡연은 비흡연과 과거에 흡연경험이 있거나 현재 흡연중인 그룹으로 분류하였고, 음주는 술을 마시지 않는 그룹과 한달에 한 번 이상 마시는 그룹으로 분류하였다. 운동실천은 유산소 신체활동 실천율을 의미하며 일주일에 중강도 신체활동을 150분 이상 또는 고강도 신체활동을 75분 이상 실천하는 경우 운동을 실천하는 것으로 분류하였다.

#### 2) 대사증후군과 위험요인

본 연구에서 대사증후군 진단은 Alberti 등[24]이 제시한 기준을 활용하였다. 단, 복부비만 기준은 아시아 전 지역에 대한 기준만 제시되어 있어, 대한비만학회에서 제시한 값을 기준으로 하였다[7]. 아래 항목 중 3개 항목 이상에 해당하는 경우 대사증후군으로 분류하였다.

① 복부비만: 복부둘레 85 cm 이상

② 혈압: 수축기 130 mmHg 또는 이완기 85 mmHg 이상

③ 고밀도지단백-콜레스테롤: 50 mg/dL 미만

④ 중성지방 150 mg/dL 이상

⑤ 공복혈당: 100 mg/dL 이상

#### 3) 자율신경활성도

**Figure 1.**
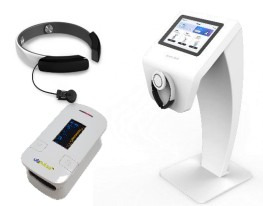
Omnifit mindcare medical device(OmniCNS, Seoul, Korea).

본 연구에서는 자율신경활성도를 측정하기 위하여 자율신경계의 기능을 반영하는 생리적 지표인 HRV를 사용하였다. HRV 검사는 안정 상태에서 5분간 심장박동을 측정한 후 정상 동성 리듬만을 대상으로 컴퓨터를 이용해 통계적으로 분석함으로써 자율신경계의 조절기능, 교감신경과 부교감신경간의 균형상태, 그리고 각각의 활성도를 평가하는 기술이다[10]. 자율신경 기능 평가를 위해 의료기기 인증(제허 13-1107호)을 받은 Omnifit mindcare medical device (OmniCNS, Seoul, Korea)을 사용하였다(Figure 1). 해당 시스템은 광용적맥파(photoplethysmography) 신호 기반의 HRV 측정을 통해 심박표준편차(standard deviation of the normal to normal interval, SDNN), 교감신경 활성도(LF), 부교감신경 활성도(HF), 자율신경 총 활성도(total power, TP) 등의 지표를 산출한다. 측정된 HRV 지표 중 시간 영역 SDNN은 millisecond 단위의 원자료를 사용하였다. 주파수 영역 LF, HF, TP는 Omnifit 시스템 내장 알고리즘을 통해 산출된 T-score (평균 50, 표준편차 10) 값을 그대로 사용하였으며, 이는 20∼60대 성인 300명의 성별 및 연령대별 평균과 표준편차를 기준으로 계산된 것이다[25].

##### (1) SDNN

SDNN은 정상 심박 간격(normal to normal interval)의 표준편차로 교감신경 및 부교감 신경의 활성도를 반영하며, 전체 자율신경 반응을 알 수 있다. 스트레스 저항도라고도 하며, 표준범위(30∼60 millisecond)내에서 점수가 클수록 자율적 조절능력이 좋은 것을 의미한다[16].

##### (2) TP

TP는 자율신경계 조절능력과 전체적인 활성도를 나타낸다. TP는 VLF, LF, HF 주파수 영역의 총 파워 값으로 계산된다.

##### (3) HF

HF는 주로 부교감신경계의 활성도를 반영한다. 주파수를 분석하여 0.15∼0.4 Hertz (Hz)의 주파수 대역이 차지하는 면적으로 측정한다. 값이 클수록 부교감신경계의 활성도가 높은 것을 의미한다.

##### (4) LF

LF는 주로 교감신경계의 활성도를 반영한다. 주파수를 분석하여 0.04∼0.15 Hertz (Hz)의 주파수 대역이 차지하는 면적으로 측정한다. 값이 클수록 교감신경계의 활성도가 높은 것을 의미한다.

### 4. 자료 수집

본 연구는 국립목포대학교 생명윤리위원회 승인(MNUIRB-250505-SB-009-01)을 받은 후 진행하였다. 자료 수집은 2023년 8월부터 2024년 12월까지 전남 장흥군에 위치한 원광대학교 장흥통합의료병원에서 일반 건강검진을 목적으로 내원하여 채혈검사, 복부둘레 측정, 자율신경기능검사를 모두 시행한 50∼65세 중년 여성의 검진자료를 대상으로 하였다. 먼저 기관장의 허락을 받고 개인정보가 모두 제거된 상태의 Comma-Separated Values 형식의 파일을 받았다. 모든 자료는 비식별화된 형태로 연구자는 개별 환자를 확인할 수 없었다. 전달받은 309명의 자료 중 문진표에 폐경으로 표시된 276명의 자료를 대상으로 최종 분석에 활용하였다.

### 5. 자료 분석

본 연구에서 수집된 자료는 SPSS version 26.0 (IBM Corp., Armonk, NY, USA)을 이용하여 다음과 같이 분석하였다. 먼저 각 변수의 정규성을 Shapiro-Wilk 검정을 통해 확인하였으며, 정규성이 충족되어 독립표본 t-검정과 Pearson 상관관계 분석을 시행하였다. 모든 검정에서 유의수준은 *p* < .05로 하였다.

1) 대상자의 대사증후군 유무에 따른 일반적 특성과 건강관련 특성은 independent two sample t-test와 Chi-square test로 분석하였다. 기대빈도가 5미만인 셀에 대해서는 Fisher’s exact test로 분석하였다.

2) 대상자의 대사증후군 유무에 따른 대사증후군 위험요인은 independent two sample t-test와 Chi-square test로 분석하였다.

3) 대상자의 대사증후군 유무에 따른 자율신경활성도 차이는 independent two sample t-test로 분석하였다.

4) 대상자의 대사증후군 위험요인과 자율신경활성도 간의 상관관계를 확인하기 위해 Pearson correlation analysis를 시행하였다.

### 6. 윤리적 고려

본 연구는 국립목포대학교 생명윤리심의위원회 승인(MNUIRB-250505-SB-009-01)을 받아 진행하였다. 본 연구자료는 기존에 수집된 이차 자료를 활용하여 수행되었으며, 자료에는 대상을 식별할 수 있는 인적 식별정보가 모두 제거된 상태로 제공받아 분석하였다. 연구 목적 이외에는 사용되지 않았으며, 연구의 전 과정에서 익명성과 기밀성이 철저히 유지되었다. 자료는 연구 종료후 3년간 안전하게 보관하며, 이후 모든 자료는 완전히 폐기한다.

## 연구 결과

### 1. 대사증후군 유무에 따른 일반적 특성과 건강관련 특성

대상자의 평균 연령은 58.06 ± 3.85세이었다. 일반적 특성으로 대졸이상이 36.6%로 가장 많았으며, 배우자가 있는 대상자는 85.9%이었다. 건강관련 특성에서는 비흡연자가 95.7%로 대부분이었고, 음주를 하지 않는 경우도 71%로 나타났다. 전체 대상자 276명 중 대사증후군은 59명으로, 유병률은 21.4%이었다. 대사증후군과 정상군 두 그룹 간의 흡연, 음주, 신체활동 모두에서 통계적으로 유의한 차이는 없었다(Table 1).

### 2. 대사증후군 유무에 따른 대사증후군 위험요인

대사증후군 유무에 따라 체질량지수, 복부둘레, 공복혈당, 수축기 및 이완기 혈압, 고밀도지단백 콜레스테롤, 중성지방 모두 유의한 차이를 보였다(*p* < .001)(Table 2).

### 3. 대사증후군 유무에 따른 자율신경활성도

자율신경활성도 중 교감신경 활성도를 나타내는 LF값이 대사증후군 군은 4.34으로 정상군의 4.69보다 유의하게 낮았다(t = 2.74, *p* = .007). SDNN, TP, HF에서는 유의한 차이가 나타나지 않았다(Table 3).

### 4. 대사증후군 위험요인과 자율신경활성도의 상관관계

공복혈당과 SDNN, TP, LF, HF 간에 각각 유의한 음의 상관관계가 있었다(r = −.16, *p* = .010; r = −.21, *p* < .001; r = −.17, *p* = .004; r = −.18, *p* = .003)(Table 4).

## 논의

본 연구에서 중년기 폐경 여성의 대사증후군 유병률은 21.4%로 나타났다. 이는 국민건강영양조사 제8기 3차년도(2021) 연구에서 폐경후 여성의 대사증후군 유병률이 25.8%[26]와 한국성인여성을 대상으로 한 연구결과[27] 24.6%와도 비슷한 수치이다. 제6기 국민건강영양조사자료를 분석한 연구에서 중년여성의 대사증후군 유병률 22.9% 중 폐경전 여성이 11%로 나타난 반면, 폐경후 초기여성에서는 22.8%, 폐경후 후기여성에서는 36.5%로 점차 증가하는 경향을 보였다[3]. 연구 결과에 따라 조금씩은 다르지만 폐경 후 여성에서 대사증후군 유병률이 높게 나타나는 것은 일관되게 보고되고 있어 폐경기 여성에서 대사증후군 관리는 더욱 중요한 시기라고 할 수 있다.

본 연구에서 폐경후 중년여성의 교육수준에 따른 대사증후군 유병률에는 차이가 없는 것으로 나타났다. 이는 교육수준이 낮을수록 대사증후군 유병률이 높게 나타난 선행연구[4]와, 교육수준이 높을수록 건강과 관련된 정보를 보다 용이하게 습득하며, 건강한 생활 습관을 유지하기 위해 노력할 가능성이 높다는 선행연구[28]와는 다른 결과이다. 이는 본 연구에서는 단순히 학력만을 기준으로 교육수준을 평가했기 때문이며, 대사증후군은 자가관리가 중요한 만큼 단순한 학력이 아니라 건강 관련한 교육에 대한 관심과 교육경험이 더 중요할 것으로 생각된다. 추후 연구에서는 단순 학력이 아닌 건강 관련 교육과 관련한 특성을 고려하여 확인해 볼 필요가 있다. 본 연구에서 결혼상태에 따른 대사증후군 유병률의 차이는 통계적으로 유의하지 않았다. 이는 폐경기 여성의 대사증후군 발생에 있어 단순한 결혼상태나 동거 여부만으로는 영향을 설명하기 어렵다는 점을 시사한다. 따라서 향후 연구에서는 식습관, 사회적지지, 정서적 요인 등 다양한 심리사회적 요인을 포괄적으로 고려한 분석이 필요할 것으로 사료된다. 음주와 흡연, 신체활동의 유무에 따라 대사증후군의 유병률의 차이는 없는 것으로 나타났는데, 폐경 후 성인여성의 대사증후군 관련요인을 살펴본 선행연구[25] 결과와 일치하였다. 신체활동은 근력운동을 일주일에 2회 이상 실천하지 않는 여성에서 대사증후군 유병률이 높았지만, 걷기 또는 유산소 신체활동의 실천여부는 대사증후군과 유의한 관련이 없는 것으로 나타났다[26,29]. 본 연구에서는 신체활동을 여가활동으로의 운동과 일 관련한 신체활동을 구분하지 않았지만 일 관련한 신체활동의 경우 복부비만과 대사적 건강지표에 부정적인 영향을 미치는 것으로[30] 보고되고 있으므로 추후에는 구분하여 분석할 필요가 있다. 또한 최근에는 신체활동의 부족과는 별개로 앉아서 보내는 시간의 과다가 비만 및 만성질환 위험도를 상승시키는 것으로 보고되고 있어[31] 폐경 후 중년기 여성에서 신체활동을 증가시키는 동시에 앉아서 보내는 시간을 감소시키기 위한 접근이 필요할 것이다.

본 연구에서 대사증후군 유무에 따른 자율신경활성도 차이에서 교감신경 활동을 반영하는 LF가 대사증후군에서 유의하게 낮았다. 대사증후군 환자들이 LF와 부교감신경을 나타내는 HF가 감소하였다고 보고한 결과[17]와 부분적으로 일치하는 결과이다. 대사증후군 그룹과 정상그룹 간의 자율신경활성도 평균값을 비교해 보았을 때 자율신경계 모든 항목에서 정상군보다 대사증후군 그룹에서 평균수치가 낮은 것으로 나타났다. 이러한 결과는 대사증후군이 있는 경우 자율신경계, 특히 교감신경의 전반적인 활성이 저하되어 있음을 시사하며 자율신경계 기능 평가가 대사질환 위험군 선별에 활용될 수 있는 가능성을 제시한다. 자율신경계활성도가 낮은 그룹에서 대사증후군 위험성이 유의하게 증가하였고[15], 인간기반 연구에서 심박표준편차를 나타내는 SDNN과 부교감신경을 나타내는 HF가 낮은 경우 심장질환 위험과 사망률을 높이는 원인 중에 하나라고 밝힌 바 있다[32]. 이는 자율신경활성도와 대사증후군 사이의 관련성을 보여주는 것으로 자율신경계의 불균형이 대사증후군의 위험을 증가시키는 요인이 될 수 있으며, 따라서 자율신경활성도가 대사증후군을 확인하는 지표로 잠재적으로 사용될 수 있다고 주장[16,32,33]한 결과를 뒷받침할 수 있다. 정상군보다 대사증후군에서 LF가 유의하게 낮은 것으로 나타난 결과는, LF 영역이 대사 항상성 유지에 필요한 자율신경계 조절 능력 저하와 밀접한 관련이 있음을 시사한다. LF 저하는 교감신경계 조절 능력의 저하를 의미할 수 있으며, 이는 혈압조절, 혈관 긴장도 등 에너지 대사 조절에 직접적 영향을 미친다[17]. 또한 LF 저하는 자율신경 불균형으로 이어져 인슐린 저항성, 혈압 상승, 내장지방 축적 등 대사증후군의 병태생리에 기여한다[34]. 그러나 본 연구에서는 HF, SDNN, TP의 차이가 통계적으로 유의하지 않았다. 이는 대사증후군 환자의 HF와 SDNN이 뚜렷하게 감소했다고 보고한 최근 체계적 문헌고찰 및 메타분석 결과[17]와 상이하다. 이러한 결과는 HRV 지표 간 민감도 및 생리적 반응 특성의 차이에 기인할 수 있다. LF는 비교적 짧은 시간 내에도 교감신경계의 활동을 민감하게 반영하여, 스트레스, 혈압변화 등과 높은 연관성을 갖는다[35]. 반면 HF는 호흡 주기에 민감하고 개별 호흡 특성에 따라 변동성이 커지므로[36], 폐경기 여성의 생리적 다양성을 충분히 반영하지 못했을 수 있다. SDNN과 TP는 장시간 측정에서 더 안정적인 해석이 가능하나[23], 본 연구에서와 같은 단시간 측정에서는 변별력이 제한적일 수 있다.

본 연구의 상관관계 분석 결과, 대사증후군 위험요인 중 공복혈당에서 자율신경활성도(SDNN, TP, LF, HF)와 유의한 음의 상관성이 나타났다. 이는 자율신경계 기능 저하가 특히 혈당 대사 이상과 밀접히 연관될 수 있음을 보여주는 근거로 해석될 수 있다. 이는 농촌 성인에서 높은 스트레스군에서 정상군보다 당뇨 유병률이 더 높다는 연구 결과와도 일치한다[37]. 공복혈당은 스트레스와 관련성이 있으며 스트레스가 높은 집단에서 고혈압, 공복혈당 및 중성지방이 유의하게 높다는 결과[38,39]를 지지하였다. 또한 대규모 인구 기반 코호트 연구인 The Maastricht Study에서는 당뇨 전단계 및 제2형 당뇨병 환자에서 HRV 지표가 정상군에 비해 유의하게 낮았으며, 공복혈당 수치가 증가할수록 SDNN, TP, LF와 뚜렷한 음의 상관관계를 보였다. 이러한 결과는 본 연구에서 확인된 공복혈당과 자율신경활성도 간의 유의한 역상관관계를 지지한다[40].

본 연구의 제한점으로는 첫째, 본 연구가 단면조사 연구에 의한 결과이므로 대사증후군과 자율신경활성도 간의 인과관계를 명확하게 추론할 수 없다는 점이다. 둘째, 단일 기관에서 실시되어 중년기 폐경 여성 전체를 충분히 대표하지 못한다는 제한이 있다.

그럼에도 불구하고 본 연구는 중년기 폐경 여성을 대상으로 대사증후군과 생행동학적 지표인 자율신경활성도 간의 관련성을 실증적으로 분석하였다는 점에서 의의가 있다. 특히 자율신경활성도는 스트레스 반응에 민감하고 반복 측정이 가능한 생체지표로, 폐경기 여성의 생리적 변화와 대사 위험요인을 보다 객관적으로 이해할 수 있는 기초자료를 제공하였다. 또한 본 연구 결과는 향후 간호중재 개발 및 적용의 이론적 기반을 마련했다는 점에서 간호학적 의의가 있다. 실제로 운동요법, 복식호흡, 명상 및 이완훈련, 마음챙김 기반 스트레스 관리 중재 등이 자율신경기능(특히 HRV 지표) 향상에 효과적임이 다수의 연구에서 보고된 바 있다. 따라서 본 연구 결과는 폐경기 여성의 자율신경 기능 저하 및 대사증후군 위험군을 조기 선별하고, 이를 개선하기 위한 맞춤형 간호중재 프로그램 설계에 실질적인 기여를 할 수 있을 것으로 기대된다.

## 결론

본 연구는 중년기 폐경 여성을 대상으로 대사증후군 위험요인과 자율신경활성도 간의 관련성을 분석하였다. 공복혈당이 SDNN, TP, LF, HF와 관련성이 높음을 확인하였으며, 또한 대사증후군에서 교감신경을 나타내는 LF 지표가 유의하게 낮아져 있음을 확인하였다. 이는 자율신경계의 기능 저하가 혈당이상과 연관되어 있으며, 대사증후군 위험군 선별과 관리에 있어 자율신경활성도 측정이 유용한 생리적 지표로 활용될 수 있음을 시사한다.

자율신경계의 기능은 가역적이며, 간호중재를 통해 회복이 가능하다는 점에서 본 연구는 간호 실무 영역에서 심신이완요법, 운동요법, 명상요법 등 비약물적 접근을 활용한 자율신경균형 증진 중재의 중요성을 뒷받침한다. 특히 폐경기 여성은 생리적, 심리적 변화가 복합적으로 나타나는 시기로, 자율신경의 건강을 증진시키는 간호중재가 대사증후군 예방에도 긍정적인 영향을 미칠 수 있다.

향후 연구에서는 다양한 연령대 및 생활환경(도시 vs. 농촌)을 포함한 비교연구, 그리고 자율신경기능 개선을 목적으로 한 간호중재의 효과성을 평가하는 실험설계 연구가 필요하다. 이를 통해 자율신경활성도를 활용한 대사증후군 위험군 선별 및 개인 맞춤형 건강관리가 가능할 것으로 기대된다.

## Figures and Tables

**Table 1.** General and Lifestyle Characteristics in the Metabolic Syndrome and Non-metabolic Syndrome Groups (N = 276)

Variables	Total (n = 276)	MS (n = 59)	Non-MS (n = 217)	t or *χ*^2^	*p*
General characteristics					
Age (years)	58.06 ± 3.85	57.71 ± 3.78	58.16 ± 3.87	0.79	.432
Education level					
≤ Elementary school	27 (9.8)	8 (13.6)	19 (8.8)	1.77	.621
Middle school	48 (17.4)	8 (13.6)	40 (18.4)		
High school	100 (36.2)	22 (37.3)	78 (35.9)		
≥ College	101 (36.6)	21 (35.5)	80 (36.9)		
Marital status					
Married, living with partner	237 (85.9)	46 (78.0)	191 (88.0)	3.86	.059
Widowed/Divorced/Single	39 (14.1)	13 (22.0)	26 (12.0)		
Lifestyle characteristics					
Smoking					
Yes (past/now)	12 (4.3)	2 (3.4)	10 (4.6)	0.17	.963^†^
No	264 (95.7)	57 (96.6)	207 (95.4)		
Alcohol consumption					
Yes	80 (29.0)	13 (22.0)	67 (30.9)	1.36	.244
No	196 (71.0)	46 (78.0)	150 (69.1)		
Exercise					
Yes	165 (59.8)	32 (54.2)	133 (61.3)	0.96	.370
No	111 (40.2)	27 (45.8)	84 (38.7)		

Values are presented as the mean ± standard deviation or n (%). MS = Metabolic syndrome; Non-MS = Non-metabolic syndrome.

† Fisher’s exact test.

**Table 2.** Metabolic Syndrome Risk Factors in the Metabolic Syndrome and Non-metabolic Syndrome Groups (N = 276)

Variables	Total (n = 276)	MS (n = 59)	Non-MS (n = 217)	t or *χ*^2^	*p*
BMI					
Normal weight	83 (30.1)	6 (10.2)	77 (35.5)	37.46	< .001
Overweight	70 (25.3)	6 (10.2)	64 (29.5)		
Obesity	123 (44.6)	47 (79.6)	76 (35.0)		
Waist circumference (cm)	83.22 ± 8.07	90.11 ± 7.52	81.35 ± 7.16	−8.25	< .001
SBP (mmHg)	122.82 ± 16.45	132.42 ± 15.76	120.21 ± 15.67	−5.30	< .001
DBP (mmHg)	67.96 ± 11.03	72.20 ± 11.18	66.81 ± 10.73	−3.39	< .001
Fasting glucose (mg/dL)	98.09 ± 14.53	109.73 ± 20.28	94.93 ± 10.55	−5.41	< .001
HDL-cholesterol (mg/dL)	59.84 ± 13.50	49.71 ± 9.75	62.59 ± 13.07	8.31	< .001
Triglyceride (mg/dL)	127.47 ± 67.13	180.69 ± 83.21	112.99 ± 53.77	−5.92	< .001

Values are presented as the mean ± standard deviation or n (%). MS = Metabolic syndrome; Non-MS = Non-metabolic syndrome; BMI = Body mass index; SBP = Systolic blood pressure; DBP = Diastolic blood pressure; HDL = High-density lipoprotein.

**Table 3.** Autonomic Nervous System Activity in the Metabolic Syndrome and Non-metabolic Syndrome Groups (N = 276)

	MS (n = 59)	Non-MS (n = 217)	t	*p*
SDNN	27.85 ± 11.38	29.68 ± 11.32	1.10	.275
Total power	5.98 ± 0.77	6.18 ± 0.72	1.77	.081
Low frequency	4.34 ± 0.86	4.69 ± 0.91	2.74	.007
High frequency	4.97 ± 0.94	5.08 ± 0.85	0.79	.432

Values are presented as the mean ± standard deviation. MS = Metabolic syndrome; Non-MS = Non-metabolic syndrome; SDNN = Standard deviation of the normal-to-normal interval.

**Table 4.** Correlations between Metabolic Syndrome Risk Factors and Autonomic Nervous System Activity (N = 276)

	SDNN		TP		LF		HF	
	r	*p*	r	*p*	r	*p*	r	*p*
Waist circumference (cm)	−.03	.584	−.05	.380	−.11	.081	−.01	.887
SBP (mmHg)	−.06	.304	−.05	.456	−.05	.374	.01	.898
DBP (mmHg)	−.02	.779	−.01	.898	−.03	.684	.06	.336
Fasting glucose (mg/dL)	−.16	.010	−.21	< .001	−.17	.004	−.18	.003
HDL-cholesterol (mg/dL)	.03	.643	.05	.371	.03	.599	.09	.160
Triglyceride (mg/dL)	.00	.967	−.05	.390	−.08	.181	−.04	.482

SDNN = Standard deviation of the normal to normal interval; TP = Total power; LF = Low frequency; HF = High frequency; SBP = Systolic blood pressure;, DBP = Diastolic blood pressure; HDL = High density lipoprotein.

## Data Availability

Please contact the corresponding author for data availability.
